# Pre-analytical barriers to blood culture completion in a large laboratory network

**DOI:** 10.1099/jmm.0.002151

**Published:** 2026-04-07

**Authors:** Timothy J. J. Inglis, Teagan F. Paton, Benjamin R. McFadden, Elizabeth Thomas, Michael J. Leung

**Affiliations:** 1 Department of Microbiology, PathWest Laboratory Medicine, PP Building, QEII Medical Centre, Nedlands, WA 6009, Australia; 2 Pathology and Laboratory Medicine, Medical School, University of Western Australia, Crawley, WA 6009, Australia; 3 Western Australian Country Health Service, Curtin University, Bentley, WA, Australia; 4 Harry Perkins Centre for Health and Medical Research, QEII Medical Centre, Nedlands, WA 6009, Australia; 5 School of Physics, Mathematics and Computing, University of Western Australia, Crawley, WA 6009, Australia; 6 School of Population Health, Curtin University, Bentley, WA, Australia

**Keywords:** blood culture (BC), bloodstream infections, incubation time, laboratory centralization, laboratory performance, quality improvement, safety and quality, transit time

## Abstract

**Introduction.** Sepsis is a major contributor to the global burden of disease. Its effective treatment is time-critical and relies on timely access to significant blood culture (BC) results.

**Hypothesis/gap statement.** The value of BC results to the requesting service is reduced by delays in report completion. Centralized clinical laboratory networks have little insight into pre-analytical causes of delayed BC results.

**Aim.** We aimed to assess variations in laboratory investigation of bloodstream infection and sepsis throughout a state-wide laboratory network, with the goal to design suitable remedial action.

**Methodology.** We analysed BCs from collection to final reporting in a public pathology service by univariate analysis and supervised machine learning.

**Results.** Of the 5,436 first-positive BCs from all Western Australian (WA) public laboratories in 2023, 1,343 (24.7%) came from regional sources. A total of 1,052 (78.3%) regional BCs were from emergency departments, and 831 (64.5%) of these were collected out of hours, rising during the 24 h cycle. Regional BCs took 33 h more than urban area cultures to reach a final report (103 compared with 70 h). Regional BC Gram stains were delayed by 31 h (69 compared with 38 h) and took over 97 h from collection to report in 25% regional Gram-stain results. Regional BCs added a 15 h delay to first results when significant species were mixed with potential contaminants, and 23 h when mixed with other significant species.

**Conclusion.** In WA, substantial delays to actionable BC results were common. The time taken to transport specimens to a laboratory was a small fraction of these delays. Monitoring of the steps in BC workflow completion can be used to improve the quality and safety of BC service provision within the limits of current technology, though solutions to this critical capability gap vary with location.

## Introduction

Despite being an imperfect measure of sepsis, blood culture (BC) remains a key to clinical sepsis management and associated antimicrobial stewardship [1]. Much effort has gone into clinical laboratory technologies that speed up BC result reports. Newer methods for rapid bacterial identification, such as multiplex nucleic acid amplification panels and matrix-assisted laser delimited interference-time of flight (MALDI-TOF) MS, reduce laboratory turnaround times, but only achieve their full potential when BCs commence their initial incubation quickly [2]. This is only possible right now in suitably equipped major centres. The earliest actionable results of BC are from Gram-stain microscopy. However, bacterial identification and susceptibility testing are more important contributors to directed therapy and clinical outcome [3]. In remote and regional Australia, sepsis management relies on traditional multi-stage, culture-dependent microbiology. The disruptive effect of the COVID-19 pandemic on pathology laboratory networks was highlighted when the shortfall in advanced diagnostic capability had to be addressed during deployment of a mobile molecular diagnostic assay to investigate a suspected major quarantine breach [4]. In the subsequent Adaptive Diagnostics for Emerging Pathogenic Threats (ADEPT) project, we addressed the need for a better-coordinated regional laboratory response for emerging infections other than COVID-19 and set out to tackle the escalating problem of Antimicrobial Resistance (AMR) sepsis in distant regional locations. Later in the pandemic, when small regional laboratories reconnected with their referral laboratory hubs, some pathology courier service providers did not re-establish a regular service to parts of regional Western Australia. A preliminary safety and quality audit revealed frequent pre-analytical delays, such as time elapsed between BC collection and culture incubation outside the minimum international standard [5]. This initial quality and safety study prompted a registered statewide quality initiative using data from calendar year 2023; the first full year after a return to pre-COVID within-state travel. The aim of the quality initiative was to assess variations in laboratory investigation of bloodstream infection and sepsis throughout a state-wide laboratory network in order to identify improvements to laboratory support for the management of sepsis in regional Australia.

## Methods

### Ethics

A clinical laboratory safety and quality audit was registered with the Health Department of Western Australia, with an emphasis on time points in the BC pathway for adult patients in the public health system during 2023. The application was approved by the PathWest Quality Improvement Committee (47343: BC workflow completion).

### Data access and protection

Data were extracted via Structured Query Language (SQL) query from the PathWest laboratory information system as comma-separated value files. Preliminary data analysis was performed using Excel (Microsoft Office, version 16.84). Identifiable data were handled on the PathWest laboratory information system computer terminals until fully de-identified, which was achieved by removing patient names and dates of birth immediately after calculating age at the time of specimen collection. To complete de-identification, patient and specimen locator codes were removed after data cleaning, so that no records could be traced back to a specific patient via their specimens.

### Data cleaning

The following BC records were removed: all records of BCs collected in the State mortuary, quality control specimens and specimens from patients younger than 16 years of age at first specimen collection. Entirely negative (no positive bottle in a BC set) cultures were excluded from further analysis. Results that precisely duplicated a first-positive culture bottle within the previous 1 month, including paired second bottles, were then removed. A manual check was performed to ensure the removal of incompletely identified subsequent isolates or variations of the same bacterial name from paired bottles. Results from paired BC bottles or subsequent sets that contained one or more different species were retained, whether deemed significant or not.

### Data labelling

The range of BC sources in the Western Australian public health system was classified into Perth metropolitan [teaching hospitals, other hospitals with 24 h emergency departments (EDs), smaller non-teaching hospitals including outlying units], regional hub hospitals known as health campuses, smaller regional hospitals with or without EDs, health centres and remote clinics. Additional columns were created to indicate whether the BC originated in a regional or Perth metropolitan area location, which region of WA, the type of microbiology laboratory service (collection centre with no laboratory, limited BC service with autoanalyser and primary subculture, or referral laboratory with MALDI-TOF, PCR-based identification including 16s sequencing), the clinical significance category of isolated micro-organism(s), BC collection in or out of hours, in the ED or not, the time of day, and day of the week collected, and the time intervals in transit from collection to incubation (time of receipt), incubation to plating out (surrogate for Gram stain) and final report release. Clinical significance was assigned entirely by bacterial or fungal species identification, without reference to the clinical context. All coagulase-negative staphylococci reported without species identification were deemed ‘Potential Contaminant(s)’, as this description was based on a more detailed assessment of clinical significance by the duty clinical microbiologist logged in the laboratory record. Thus, all coagulase-negative staphylococci identified to species level were reported as potentially significant. Categories used were single significant species (SSS), more than one (multiple) significant species, significant species mixed with possible contaminant, more than one (multiple) possible contaminant species, single possible contaminant species and indeterminant, usually due to no growth on subculture. After data labelling was complete, a de-identified master file was produced and used for subsequent analysis.

### Statistical analysis

Univariate analysis was conducted in Excel (Microsoft Office). Accurate specimen counts and data extraction for defined subsets from the master file were completed using a pipeline developed using the Python programming language (v 3.12.4), including the Pandas (v 2.2.2) and Numpy (v 1.26.4) packages. All further analysis was carried out with Prism, v10.2.3 (GraphPad, San Diego, CA, USA), and comprised column statistics, frequency histograms, t-tests, life tables and multivariate combinations.

### Dimensional reduction

The de-identified master data file was then imported into an open-source machine learning package (ORANGE data mining v 3.38.0, University of Ljubljana, Slovenia), which was used to pre-label and visualize the statewide positive BC data set, and its regional subset with t-stochastic neighbourhood embedding (t-SNE) using all 30 variables, and radial visualization (RadViz) to display the combination of four workflow completion time variables against two of the remaining features.

## Results

### Services generating BCs

**Fig. 1.**
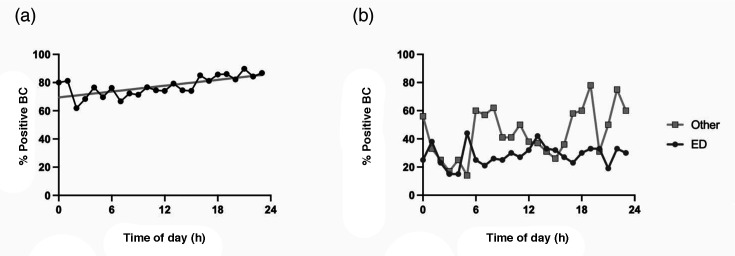
(**a**) Percentage of Positive BCs collected in regional EDs by Time of day. The regression line slope is significantly different from zero (*P*=0.0002). (**b**) The percentage of Positive regional BCs containing potential contaminant species byTime of day, comparing ED cultures with all other regional sources. ED median=28.5%, other median=41. Mann–Whitney U test: U=129, *P*=0.0007.

Of 5,432 BC sets, 1,343 positive sets (24.7%) were from regional centres, under-representing the 28.6% of the WA population residing in the regions (Fisher’s Exact, *P*<0.0001). A total of 1,052 of these (78.3%) were from regional EDs, 291 (21.6%) were from other hospital sources and collection centres, and only 7 (0.5%) were specifically attributed to regional general practices (Table 1) (Table 2), none of which contained mixed species. Six hundred seventy-nine (64.5%) of the 1,052 ED BCs were collected out of hours (nights, weekends and public holidays), compared with 152 from other regional hospital sources or 291 from collection centres (Fisher’s Exact test, *P*=0.0002). Five hundred thirty-one (50.5%) of all regional positive BCs came from EDs out of hours. The hourly proportion of positive BCs from EDs rose steadily over the 24 h cycle, from 61.9% (13 BC sets at 0200 h) to 80% (53 BC sets at 2100 h) (Fig. 1a). The proportion of BCs from EDs containing potential contaminants remained lower than all other sources for most of the 24 h cycle (Fig. 1b; Mann–Whitney U test, *P*=0.0007).

**Table 1.** BC pathway times

BC collected	*n*	Transit (h)^*^	Gram (h)	Final (h)	Significance^†^
Perth Metro	4,090	2.7^‡^ (1.6–4.8)^§^	38 (24–55)	70 (54–96)	
Regional	1,343	4.9 (0.4–14)	69 (46–96)	103 (74–145)	*P*=0.0009; <0.0001; <0.0001
In hours	512	3.3 (0.38–8.7)	69 (46–95)	104 (75–146)	
Out of hours	831	5.7 (0.4–18)	70 (46–96)	102 (74–144)	*P*=0.0007; ns^||^; ns
Emergency	1,052	3.9 (0.37–13)	69 (45–95)	103 (74–144)	
Other	291	6.9 (0.95–17)	70 (48–97)	102 (75–148)	*P*<0.0001; ns; ns

*Time in hours from BC collection to incubation (transit); collection to Gram stain (Gram); collection to final report (final). †Mann–Whitney U test, *P*-value. ‡Median. §Interquartile range. ||Not significant.

**Table 2.** BC processing location

BC process location	*n*	Transit (h)*	Gram (h)	Final (h)
Regional				
On-site	729	0.45 (0.2–1.6)	86 (68–97)	123 (97–169)
Off-site	546	14 (6.7–22)	46 (38–68)	80 (66–144)
Significance		*P*<0.0001	*P*<0.0001	*P*<0.0001
Perth Metro				
On-site	2,291	1.9^‡^ (1.1–2.2)^§^	42 (30–61)	73 (61–101)
Off-site	1,802	4.8 (3.5–8.3)	31 (21–47)	64 (51–89)
Significance		*P*<0.0001	*P*<0.0001	*P*<0.0001

*Hours; time to incubation (transit); incubation to Gram stain (Gram); time to final report (final). †Mann–Whitney U test, *P*-value. ‡Median. §Interquartile range. ||Not applicable.

### BC workflow completion times

Time to BC incubation, preliminary microscopy and final report were all longer for regional BCs than for the reference-centre standard BCs; BCs processed in a major laboratory on the same site as specimen collection (Table 3). The median time difference to actionable results for SSS was an increase of 3.4 h in transit, 31 h to Gram stain and 2 h to final report. In every isolate significance category, the time to preliminary actionable BC results was greater than the reference-centre standard by 24 h or more, and the difference was 25 h or more to final regional BC reports. The presence of additional species in a BC with a significant isolate added a median of 23 h if mixed significant species and 15 h if mixed significant and potential contaminant species. The increased regional specimen transit times were a small proportion of the time to Gram stain in all significance categories. Potential contaminants were present in 36.2% of positive BCs from regional sources.

**Table 3.** Isolate significance

Significance*	*n*	Transit (h)	Gram (h)	Final (h)
Regional BCs				
SSS	765	5.2^†^ (0.42–14)^‡^	69 (44–94)	71 (54–106)
MSS	79	3.2 (0.38–15)	92 (70–121)	145 (93–176)
SSPC	51	5.2 (0.25–16)	84 (64–118)	122 (89–166)
SPC	344	3.7 (0.37–12)	62 (46–90)	94 (72–140)
MPC	91	6.4 (0.5–15)	62 (43–90)	95 (71–126)
Ind.	13	1.0 (0.22–1.2)	125 (76–146)	165 (110–357)
Perth Metro				
SSS on-site^§^	1,291	1.8 (1.0–2.3)	38 (26–51)	69 (56–91)

*SSS, MSS, SSPC, SPC, MPC and Ind., usually due to no growth on subculture. †Median. ‡Interquartile range. §BC processing on same hospital campus as specimen collection in laboratory equipped with MALDI-TOF mass spectrometer and advances antimicrobial susceptibility testing.

Ind, indeterminant ; MPC, more than one (multiple) possible contaminant species; MSS, more than one/multiple significant species; SPC, single possible contaminant species; SSPC, significant species mixed with possible contaminant.

### Regional variation in BC generation

**Fig. 2.**
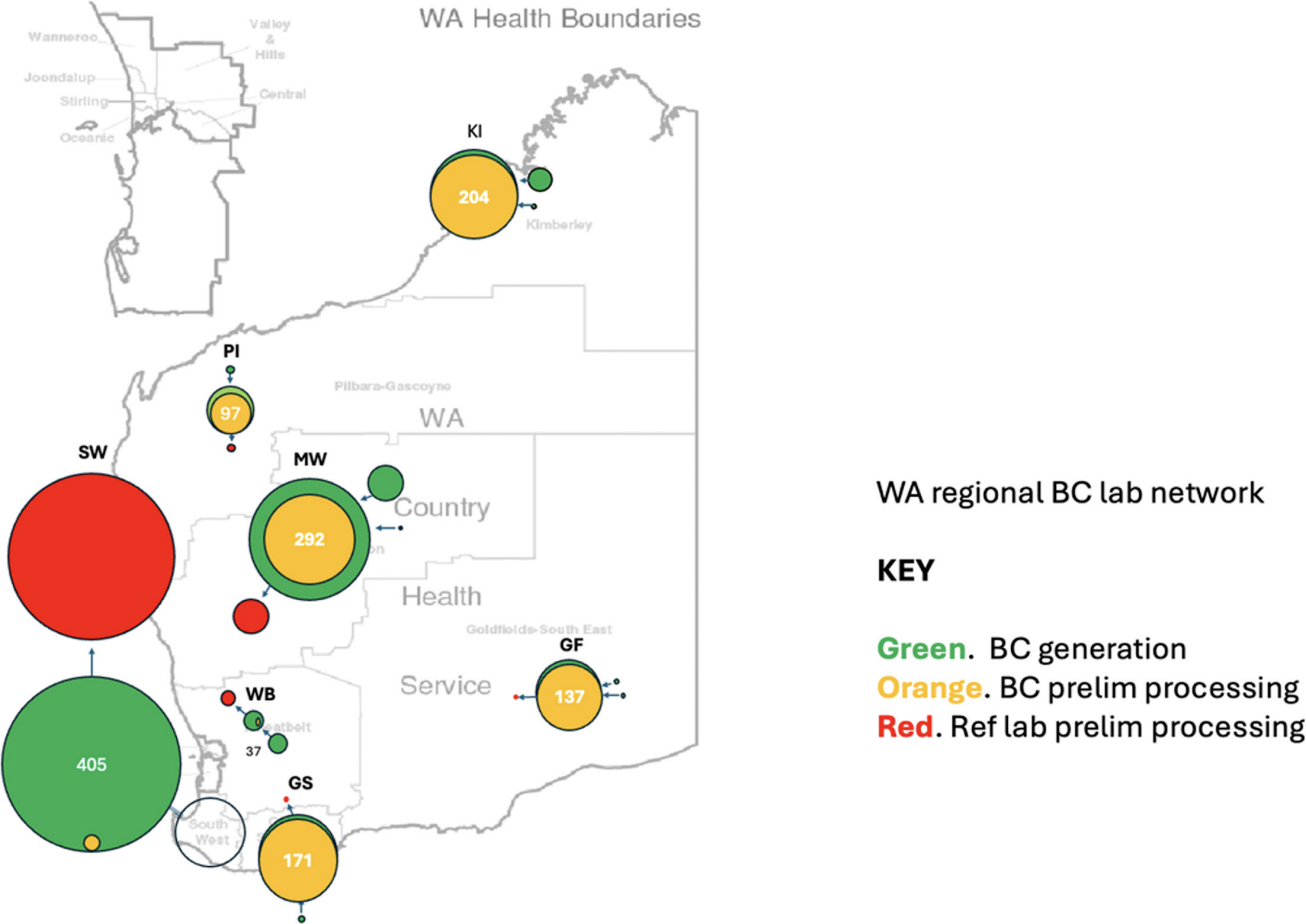
Western Australian regional BC referral network showing the relative scale of positive BC sources (green), preliminary processing (orange) and referral for definitive identification and susceptibility testing (red). The relative proximity of the Southwest region to the Perth Metropolitan area and availability of frequent road courier services support BC referral for all processing. Reliance on air services to transport specimens from other regions forces greater reliance on local laboratory services for preliminary processing. Regional abbreviations: KI, Kimberley; PI, Pilbara; SW, South West; MW, Mid West; GF, Goldfields; WB, Wheatbelt; GS, Great Southern.

The regional distribution of positive BC workload during 2023 was uneven, even allowing for differences in population density through WA (Fig. 2). The busiest regional source of positive BCs was the Southwest region, which includes WA’s largest regional city, Bunbury. The one remaining centre with on-site BC incubation at the start of 2023 in the Southwest region converted to off-site BC processing at a referral laboratory in June 2023. After that, no BCs were incubated and processed on-site in the entire region for the rest of the year.

## Discussion

Timely generation of actionable BC results is the aim of any BC service, yet all too often results reach the requesting clinician too late to influence antimicrobial therapy decisions. In this statewide audit of pre-analytical factors affecting time to complete BCs, we identified multiple contributors to delayed results. BC relies on repeated growth cycles to amplify a starter culture to reach a detection threshold. The standard shortcuts to expedite a final laboratory report are bacterial identification by MALDI-TOF mass spectrophotometer analysis performed directly on analyser-positive BC [3], multiplex nucleic acid amplification panels [2] and calibrated direct susceptibility tests [6]. Though technically feasible in regional hub laboratories [7], the skilled staff and infrastructure needed to sustain these accelerated BC methods can only be maintained in large central laboratories. In the current study, a highly centralized service operating a hub-and-spoke laboratory network served the world’s largest subnational public health jurisdiction and delivered BC results after a median of 38 h in the Perth metropolitan area and a median of 69 h in regional centres. The difference in median time to the first actionable result of Gram-stain results is therefore 31 h, or almost double the time from BC collection in the Perth metropolitan area.

**Fig. 3.**
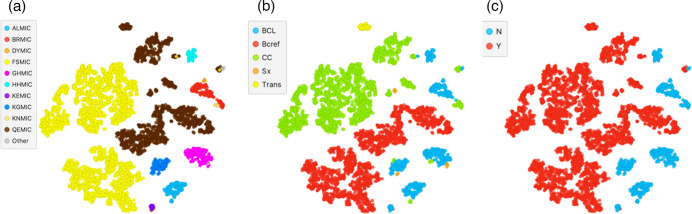
t-SNE depiction of the multidimensional (30 dimensions) relationship. (a) The main urban and regional laboratories, (b) the types of BC service and (c) the availability of MALDI-TOF on-site. KEY: (a) FSMIC, QEMIC and KEMIC are in Perth, WA; all others are in regional WA. (b) BCL, BC-enabled laboratory; Bcref, BC reference laboratory; CC, BC collection centre; Sx, general practice; Trans, regional laboratory transitioning from BCL to CC. (c) N: no MALDI-TOF, Y: MALDI-TOF-enabled.

**Fig. 4.**
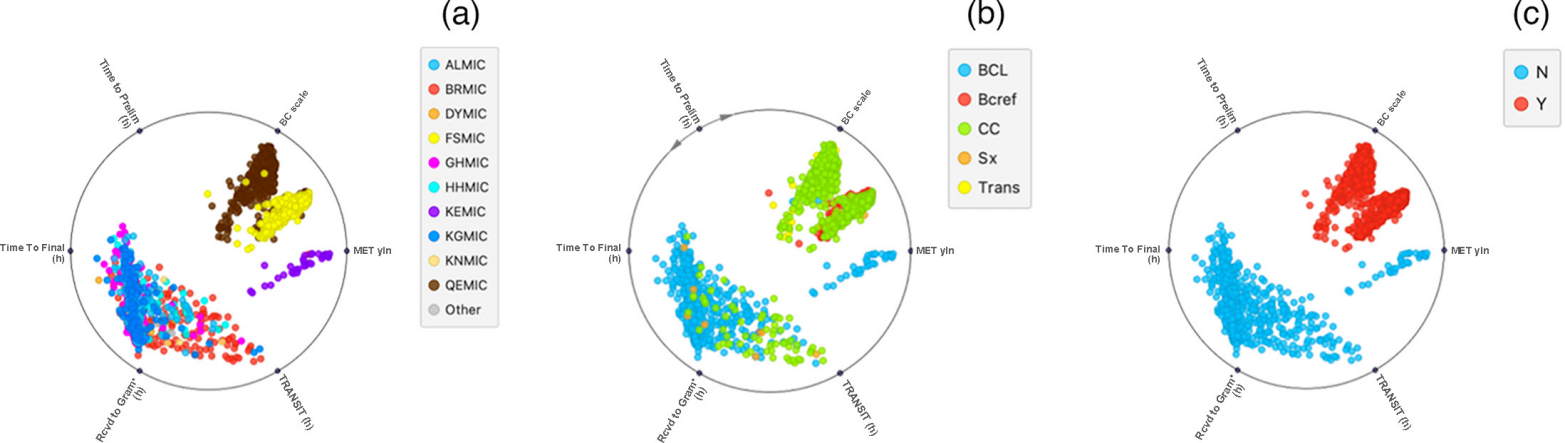
RadViz display showing BC workflow completion time features against regional origin and BC laboratory service scale. (a) The top 10 clinical laboratories by positive BC workflow, (b) the level of BC service support and (c) the availability of rapid MALDI-TOF BC isolate identification (in two large laboratories only). The time-interval axes (5–11 o'clock) separate out the regional branch laboratory-processed positive BC cluster from the Perth metropolitan clusters (KEY: same colourization scheme as in Fig. 3). N: no MALDI-TOF, Y: MALDI-TOF-enabled. BCL, BC-enabled laboratory; Bcref, BC reference laboratory; CC, BC collection centre; Sx, general practice; Trans, regional laboratory transitioning from BCL to CC.

Regions in the north of the state that are equipped to incubate BCs had a relatively high workload, but still had to refer specimens onwards to a larger referral laboratory in Perth for definitive confirmatory testing. Some collection centres in the Kimberley region are so remote that lengthy onward specimen transfer was needed before commencing initial BC processing by regional branch laboratories such as Broome, Derby and Kununurra. At the other end of the spectrum was the large Wheatbelt region, served mainly by road courier transfer to Perth and a single BC autoincubator-enabled laboratory. The different t-SNE colourizations in Fig. 3 show the complexity of BC handling in WA. Fig. 4 is a RadViz of BC handling clusters based on principal component analysis.

Centralized processing of BCs from WA’s entire Southwest region translated into lengthy transit by road courier and prolonged transport times more than international minimum standards (4 h). However, lengthy transport times did not explain the size of the discrepancy between specimens from patients in WA teaching hospitals versus regional centres. In the Southwest region, the trade-off between transit time and faster actionable BC results favoured off-site BC processing [5]. The logistic consequences of the complex multi-stage processing of BC adversely impacted other parts of regional WA, where the distances between remote clinics, regional centres and the central referral laboratories, co-located with the two largest teaching hospital centres, ruled out a road courier solution.

A decade ago, multiplex nucleic acid amplification BC identification panels were successfully trialled in regional WA [7]. Despite that work and subsequent experience running Severe Acute Respiratory Syndrome coronavirus 2 (SARS-CoV-2) molecular assays in regional centres, molecular BC identification assays have not yet been introduced to regional WA. Overly cautious urban health administration and a failure to agree on where in the value chain the service cost should sit might be behind this delayed implementation, but should not prevent dealing with a capability gap that affects thousands of patients per year.

In the absence of a one-size-fits-all solution to delayed clinical laboratory support in bloodstream infections, this audit suggests measures that would better support frontline clinical staff managing regional and remote patients with suspected bloodstream infection (Table 4). As the majority of these patients present in EDs, forward placement of BC incubators [8], culture collection best-practice training for frontline clinical staff [9] and BC timeline performance feedback [10] should be concentrated in support of EDs and practitioners who refer suspected sepsis and bloodstream infections (SABI) to EDs. Higher rates of potential BC contamination in collection centres and non-ED hospital wards require more detailed analysis and a tailored approach to time- and place-specific drivers of BC contamination risk. More consistent time point recording is needed for key decision points in the BC workflow, from collection onwards. Laboratory information systems already collect these data for internal audit purposes [5]. Business intelligence software tools permit analysis and visualization of aggregate data that can be readily digested by end users. It is our view that the aggregate BC workflow performance data should be provided as a real-time audit to frontline health service providers as part of a wider safety and quality improvement process for SABI management in regional Australia.

**Table 4.** Potential improvements to regional BC services

Pre-analytical	In-laboratory	Post-analytical
Enable BC collection by all frontline clinical staff	BC incubator notification alarm for out-of-hours positives	Provide periodic summaries of BC workflow completion audit to principal service users
Concentrate support on EDs	Continuous monitoring of BC workflow time points	Continuous monitoring of BC completion for requesters
Install BC incubators in EDs	Real-time audit of outlier-delayed results	Quality and safety review reports on BC completion outliers
Expedited BC transport when a sepsis code is activated	Record times of incubator alert, Gram-stain completion and report to clinical service	Targeted, service-specific guidance on procedure, indications and timeline progression profile
Avoid 'routine' BC on phlebotomy round	Blind subculture of outlier-delayed-entry BCs	Big data analysis of delayed BC workflow completion hotspots
Incentivize contamination avoidance during BC collection	Establish multiplex BC identification capability in regional hub laboratories	
Centralize incubation where a road courier service exists		

In conclusion, this state-wide safety and quality audit of BC completion revealed substantial delays in turnaround time for regional patients, with these samples taking 1–2 days longer to generate actionable data than those from metropolitan teaching hospitals. The clinical value of initial BC results rapidly diminishes in most patients more than 48 h after specimen collection. As the majority of positive BCs are collected out of hours and in EDs, quality improvement initiatives must prioritize these clinical settings. Widely accessible business intelligence tools could be used to map the time-, location- and service-specific drivers of BC delays throughout the health system. This is an equity issue that deserves urgent attention matched by a commitment to remedial action.

## Abbreviations

ADEPT: Adaptive Diagnostics for Emerging Pathogenic Threats
AMR: antimicrobial resistance
BC: blood culture
COVID-19: coronavirus disease 2019
EDs: emergency departments
MALDI-TOF: matrix-assisted laser delimited interference-time of flight
RadViz: radial visualization
SABI: sepsis and bloodstream infection
SARS-CoV-2: severe acute respiratory syndrome coronavirus 2
SQL: structured query language
SSS: single significant species
t-SNE: t-stochastic neighbourhood embedding
WA: Western Australia
